# Methods of Advanced Wound Management for Care of Combined Traumatic and Chemical Warfare Injuries

**Published:** 2008-07-21

**Authors:** John S. Graham, Travis W. Gerlach, Thomas P. Logan, James P. Bonar, Richard J. Fugo, Robyn B. Lee, Matthew A. Coatsworth

**Affiliations:** ^a^ Medical Toxicology Branch, US Army Medical Research Institute of Chemical Defense, Aberdeen Proving Ground, MD; ^b^ Department of Surgery, David Grant Medical Center, Travis Air Force Base, CA; ^c^ Medical Diagnostic and Chemical Branch, US Army Medical Research Institute of Chemical Defense, Aberdeen Proving Ground, MD; ^d^ Investigations Branch, US Air Force School of Aerospace Medicine/FEH, Brooks City-Base, TX; ^e^ MediSURG Research and Management Corporation, Norristown, PA; ^f^ S3 Program, Strategies, and Operations Office, US Army Medical Research Institute of Chemical Defense, Aberdeen Proving Ground, MD; ^g^ Office of the Command Surgeon, Headquarters Air Force Special Operations Command, Hurlburt Field, FL

## Abstract

**Objective:** Chemical warfare agents are potential threats to military personnel and civilians. The potential for associated traumatic injuries is significant. Damage control surgery could expose medical personnel to agents contaminating the wounds. The objectives of this study were to demonstrate efficacy of surgical decontamination and assess exposure risk to attending personnel. **Methods:** Weanling pigs were randomly assigned to 2 of 4 debridement tools (scalpel, Bovie^®^ knife, Fugo Blade^®^, and Versajet^™^ Hydrosurgery System). Penetrating traumatic wounds were created over the shoulder and thigh and then exposed to liquid sulfur mustard (HD) for 60 minutes. Excisional debridement of the injuries was performed while vapors over each site were collected. Gas chromatography was used to measure HD in samples of collected vapors. Unbound HD was quantified in presurgical wound swabs, excised tissues, and peripheral tissue biopsies following solvent extraction. **Results:** Excisional debridement produced agent-free wound beds (surgical decontamination). A significant amount of HD vapor was detected above the surgical fields with each tool. Apart from the Versajet^™^ producing significantly lower levels of HD detected over thigh wounds compared with those treated using the scalpel, there were no differences in the amount of agent detected among the tools. All measured levels significantly exceeded established safety limits. Vesicating levels of unbound HD were extracted from excised tissue. There was no measured lateral spreading of HD beyond the surgical margins. **Conclusions:** There is significant occupational exposure risk to HD during surgical procedures designed to stabilize agent-contaminated wounds. If appropriate protective measures are taken, surgical decontamination is both effective and safe.

Chemical warfare agents (CWAs) are potential threats to both military personnel and civilians. The potential of associated traumatic injuries requiring surgical treatment is significant, especially in a battlefield scenario. An existing traumatic wound may be contaminated with CWAs following the release of vapor or liquid droplets from chemical storage cylinders or their dispersal via aerial sprays, chemical bombs, or mortar shells. Exploding chemical munitions may, in addition, inflict traumatic wounds that become contaminated with agent, concurrently inflicting a combined injury. Liquid contamination of skin can also occur on contact with contaminated surfaces. The standard of care in today's casualty management system provides damage control surgery within the battlefield arena to stabilize traumatic injuries before transportation to upper echelon medical facilities. Such surgery and patient movement could potentially expose medical personnel to CWAs that are contaminating the wound. To date, there are no officially sanctioned modalities to decontaminate wounds besides 0.5% hypochlorite solution irrigation and lavage, which has relative contraindications to its use in trauma patients. There are also no standardized or optimized methods of management for casualties with combined chemical and traumatic wounds that prevent or minimize surgical team exposure while treatment is rendered.

Following the terrorist attacks on September 11, 2001, and as a result of battlefield experiences during operations Desert Shield and Desert Storm in 1992, the modern theater trauma system was matured by May 2004.^1^ Early far-forward surgical intervention, in relatively close proximity to combat operations,^2^ is required for saving lives on the battlefield. Those who need to be treated include military personnel, coalition forces, civilian contractors, local nationals (including civilians and security forces), and enemy combatants.^2^,^8^ During Desert Storm and the early phases of Operation Iraqi Freedom, delays of greater than 4 hours in transport to traditional military surgical units increased the risk of death from exsanguination, indicating a need for more proximate trauma surgical capability.^2^ The tiered military system now in place includes the following trauma center levels: Echelon I (Emergency Medical Services personnel, corpsmen, or medics); Echelon IIA (battalion aid stations and outpatient clinics); Echelon IIB (forward surgical teams); Echelon III (theater hospitals or regional trauma centers); Echelon IV (large nontheater hospitals); and Echelon V (major US-based military hospitals).^1^ The combat support hospitals (CSH; Echelon III) and forward surgery teams, including the Army forward surgery teams, Marine forward resuscitative surgery suites, and the Air Force expeditionary medical support units, provide far-forward tactical surgical intervention.^1^,^13^ These facilities typically do not provide definitive surgical care but rather damage control surgery to impact mortality and morbidity and maximize the potential for limb salvage by early intervention.^7^ Their *primary goals* are to control hemorrhage and contamination and to avoid the lethal triad of hypothermia, acidosis, and coagulopathy.^6^,^12^ The most common operative procedures conducted in these units are fasciotomies, laparotomies, craniotomies, wound debridement, soft tissue stabilization, completion of amputations, restoration of blood flow via repair or temporary vascular shunting, rapid external fixation or splinting of extremity injuries, orthopedic and ophthalmologic procedures, major vascular procedures, face- and neck-related procedures, and thoracic procedures.^4^,^11^,^12^ The forward surgery teams are typically used during the assault phase of operations^7^ and used only if flight times are longer than 90 to 120 minutes from a CSH.^12^ After initial resuscitation and stabilization, patients are transported to a higher level of care as soon as possible (eg, Landstuhl Regional Medical Center, Landstuhl, Germany).^9^ Military patients are eventually transported back to the United States to a military facility such as the Brooke Army Medical Center (Fort Sam Houston, Tex); Walter Reed Army Medical Center (Washington, DC); and the National Naval Medical Center (Bethesda, Md).^9^ Wounded military personnel can make it to an intensive care unit in the United States within 2 to 4 days of injury from the farthest reaches of Iraq or Afghanistan with the efforts of the US Air Force critical care air transport teams.^12^,^13^

The *primary purpose* of this study was to test the concept of “surgical decontamination” for a selected CWA with delayed onset of effects. An agent that delivers its effect over time and can persist within a wound, such as a mustard, could conceivably be surgically removed to effect cure or at least mitigate injury, while protecting personnel within the casualty care system. The tested hypothesis was that a wound contaminated with sulfur mustard (2,2′-dichlorodiethyl sulfide; HD) could be thoroughly decontaminated through excisional debridement despite delay in treatment. A key feature of this test was to ascertain the degree of lateral contamination peripheral to the wounded area along subcutaneous fat or fascial planes. The hypothesis was tested using an experimental procedure that approximated the initial phase of combat wound management. The *second purpose* of this study was to assess the hazard posed to attending medical personnel by measuring the amount of HD vapor generated during the excisional debridement of soft tissue wounds after 60 minutes of HD liquid exposure, using standard and innovative strategies employed in the surgical treatment of traumatic wounds. The tools used for the excisional debridement of wounds in this study included a no. 15 scalpel blade (The Kendall Company, Mansfield, Mass); a Bovie^®^ electrosurgical knife (Aaron 1250, Aaron Medical, Saint Petersburg, Fla); the Fugo Blade^®^ M100 anterior capsulotomy unit (MediSURG Research and Management Corporation, Norristown, Pa); and the Versajet^™^ hydrosurgery system (Smith & Nephew Inc, Largo, Fla).

## MATERIALS AND METHODS

### Animal model

Twelve female Yorkshire crossbred pigs (weanlings), *Sus scrofa*, 11 to 14 kg (mean = 13 kg), were used (Country View Farms, Shanksville, Pennsylvania). They were quarantined on arrival for 7 days and screened for evidence of disease before use. They were maintained under an animal care and use program accredited by the Association for Assessment and Accreditation of Laboratory Animal Care International. The Institutional Animal Care and Use Committee at the US Army Medical Research Institute of Chemical Defense, Aberdeen Proving Ground, Maryland, approved the experimental protocol. Animals were supplied tap water ad libitum and fed approximately 600 g of Harlan Teklad Miniswine Diet (W) no. 8753 (Harlan Teklad, Madison, Wis) twice a day. Animals were housed individually in 4 × 6-ft pens with coated expanded metal floors. The cages allowed visual, auditory, and olfactory contact with conspecifics. The room was maintained at 21°C ± 2°C with 50% ±10% relative humidity using at least 10 complete air changes per hour of 100% conditioned fresh air. Animal rooms were maintained on a 12-hour light/dark full-spectrum lighting cycle with no twilight. Feed was withheld for 12 hours before anesthesia. It should be noted that pigs, in general, are the best laboratory models for dermatological research for humans. The histological characteristics of pig and human skin are comparable and display similarities in epidermal thickness and composition, epidermal enzyme patterns, epidermal tissue turnover time, lipid content, character of keratinous proteins, antigenicity, pelage density and pattern of hair growth, dermal structure, deposition of subdermal fat, and general morphology.^14^,^25^

### Surgical instruments used in excisional debridements

Surgical steel in the form of a no. 15 scalpel blade was combined with direct pressure and ligature for hemostasis during debridement.

The Bovie^®^ electrosurgical knife is a multipurpose electrocautery device used as a soft tissue cutting tool and for cauterizing bleeders. It features both monopolar and bipolar functions and was utilized in this study in the coagulation mode with the power setting at 30 W. It was equipped with a standard spatulated blade. This technology and the surgical scalpel are the most prevalent surgical technologies used in military medicine.

The Fugo Blade^®^ M100 anterior capsulotomy unit, approved by the Food and Drug Administration (FDA) for anterior capsulotomy procedures conducted during cataract surgery, transciliary filtration, and peripheral iridotomy, is a radiofrequency electrosurgical incising instrument^26^,^31^ that uses little power and produces no heat. The instrument focuses a low-power energy field into a 50-µ m-wide column of plasma energy. This energy field ablates bonds of biological molecules and creates very little smoke debris. This technology has demonstrated low-power microbe decontamination of live tissue surgical sites. The instrument induces no concussion and provides noncauterizing hemostasis called “autostasis.”^32^ Histologic studies at the University of South Carolina and Louisiana State University have demonstrated that the Fugo Blade^®^ cuts in a resistance-free fashion and leaves extraordinarily clean incision walls that are similar to an ablation path of an excimer laser.^27^,^30^,^33^ Biomechanics of incision rim strength were characterized at the University of South Carolina,^34^ demonstrating that the Fugo Blade^®^ produces an incision in fragile tissue, that is, almost twice as strong as that produced by diathermy.^34^ Generally speaking, stronger incision margins translate into healthier wounds that should heal faster with fewer complications. These product attributes led to the Fugo Blade's^®^ inclusion in this study. As it is rated for microscopic surgical procedures only, with a maximum cut depth rating of 700 µ m, the Fugo Blade^®^ M100 unit was set to its maximum power output with the manufacturer's concurrence to accomplish the larger cut depths required by this study. A macroscopic surgical Fugo Blade^®^ system is being developed because this technology has promise as a battery-operated, ultra-lightweight, operator-worn medical cutting tool for use in far-forward resuscitative surgery and personnel rescue.

The Versajet^™^ hydrosurgery system is an FDA-approved instrument that generates a high-velocity waterjet, which provides efficient debridement and cleansing of traumatic wounds (including burns), chronic wounds, and other soft tissue.^35^,^44^ It is typically used in applications that would require the use of a pulse lavage device with sharp debridement. The design and location of the evacuation tube in the hand piece create a vacuum that provides efficient and safe removal of debris, tissue, fluids, and contaminants. With sufficient pressure, this tool can also cut through skin, muscle, and cartilage. This instrument was used within its designed parameters.

### Anesthesia

Before the procedure, each animal was anesthetized by intramuscular injections of xylazine HCl (Xyla-Ject^®^, Phoenix Pharmaceutical Inc, Saint Joseph, Mo; 0.5 mL @ 20 mg/mL) and a combination of tiletamine HCl and zolazepam HCl (Telazol^®^, Fort Dodge Animal Health, Fort Dodge, Iowa; reconstituted to 100 mg/mL and 0.5 mL administered), and hair removed from the dorsal and ventral surface with an electric clipper. Orotracheal intubation was performed using a 4.5 French endotracheal tube under direct visualization. The animals were placed in a chemical fume hood onto a stable platform in the left lateral decubitus position. A therapeutic heating pad (Gaymar Industries Inc, Orchard Park, New York), with the circulating water temperature set at 41°C, was placed under the animal during the exposure period to minimize hypothermia. The animals were then administered 1.0% to 2.5% isoflurane (AErrane^™^, Baxter Pharmaceutical Products Inc, Deerfield, Ill) in oxygen at a flow rate of 0.6 to 1.0 L/min using an MDS Matrix Spartan VHC anesthesia machine (Matrix, Orchard Park, New York) equipped with a Matrix VIP 3000 isoflurane calibrated vaporizer (Matrix, Orchard Park, New York). Body temperature, heart rate, respiratory rate, and oxygen saturation were monitored throughout each procedure by using a Surgivet TPR monitor (Surgivet, Waukesha, Wis). After the experimental procedures were completed, the animals were euthanized with an overdose of pentobarbital sodium euthanasia solution (Fatal-Plus Solution^®^; 78 mg/kg IV, Baxter Pharmaceutical Products Inc, Deerfield, Ill).

### Sulfur mustard

The HD employed in this study was lot HD-U-2325-CTF-N-1, 97.2 mol% (US Army Research, Development and Engineering Command, Aberdeen Proving Ground, Md). Stock solutions of HD in absolute alcohol at 9.5 mg/mL were diluted with hexane to 1.9 ng/µ *L* for the calibration standards used in gas chromatography (GC) analyses.

### Wound generation and sulfur mustard exposure

The right side of each animal was draped with plastic-backed absorbent paper. A plastic wound template was used to mark areas over the right shoulder and thigh for even spacing and positioning of the penetrating traumatic wounds and for marking the location where peripheral biopsies were to be collected along surgical margins (Fig 1a). Each wound (1 on the shoulder and 1 on the thigh of each animal) consisted of 5 full-thickness punch biopsies evenly spaced within a 4-cm diameter circle using 10-mm diameter surgical punches (Acu-Punch Skin Biopsy Punch, Acuderm Inc, Fort Lauderdale, Fla) that penetrated fascia down to underlying muscle. A dry 4″ × 4″ gauze dressing was applied with direct pressure for 5 minutes to achieve hemostasis. Following the biopsy procedure, tissues from 4 of the 5 biopsies were placed together as negative controls into 50 mL of 99.9% *n*-hexane for HD extraction.

Using an Eppendorf repeating pipettor (Eppendorf North America Westbury, New York), 200 µ *L* of undiluted HD was placed into each of the 5 biopsy cavities of each animal's wounds. The total amount of HD applied to each wound was 1.24 g (1.24 g/mL × 0.2 mL/cavity × 5 cavities/wound = 1.24 g/wound). The HD remained in the wounds for a total dwell time of 60 minutes to allow sufficient time to saturate the wound and mimic the average duration of time needed to evacuate a casualty to the nearest forward surgery team or CSH. Immediately following the 60-minute interval, the residual mustard was removed using 6 absorbent cotton-tipped swabs per wound; the swab tips were then removed and placed together into a container with 50 mL of 99.9% *n*-hexane for HD extraction. No decontaminant was used on the wounds.

### Excision procedures

At the beginning of each surgical procedure, a rectangular (4.5 cm × 4.5 cm) area of skin centered over the wound was incised down to the subcutaneous tissue, using a no. 15 blade scalpel. The skin within this demarcated area was then excised using 1 of 4 surgical instruments: a no. 15 scalpel blade (Fig 1b), a Bovie^®^ electrosurgical knife (Fig 1c), the Fugo Blade^®^ M100 anterior capsulotomy unit (Fig. 1d), or the Versajet^™^ hydrosurgery system (Fig. 1e). Animals were randomly assigned to 2 of the 4 surgical tools (1 per wound). The Bovie^®^ pad and hand piece were decontaminated and disposed of (in accordance with regulations governing disposal of HD-contaminated waste) after each excision. The Fugo Blade^®^ was set on high-power mode with the intensity set on 10 of 10 and utilized a sharp-angled tip. Before each use, the hand piece was wrapped in a disposable plastic sheath to protect the instrument from HD contamination. The Versajet^™^ was set on high (power setting of 10 of 10). The hand piece with a 15°, 14-mm head was chosen because of the tangential nature of the excision. Five hundred milliliters of lactated Ringer's solution was used as the cutting/irrigating solution for each procedure. Each hand piece was decontaminated and disposed of after each excision.

Each excision was performed over a 2-minute period. A dry 4″ × 4″ gauze was placed over the excised wound bed to minimize bleeding. The excised specimen was placed on a precut plastic-backed absorbent paper and cut into 9 pieces of equivalent size; the pieces were placed together directly into 50 mL of 99.9% *n*-hexane for HD extraction. Punch biopsies were then excised 2 cm distant from each of the 4 surgical margins down to and including the underlying fascia and a portion of underlying muscle, using an 8-mm surgical punch. The 4 biopsies were placed together into 50 mL of 99.9% *n*-hexane for HD extraction. Using a clean set of instruments, the same procedure was performed excising 4 biopsies immediately adjacent to the surgical margins, which were also placed together into 50 mL of 99.9% *n*-hexane for HD extraction. Following all biopsy procedures, the animals were euthanized as described above and the skin surrounding both wounds was widely excised using a no. 15 scalpel blade and then decontaminated and disposed.

### Minicams^™^ measurements

Vapors over each wound were collected during the entire 2-minute-excision period and the collected fumes were monitored for the presence of HD. The vaporized gases were collected with a 147-mm-diameter glass Pyrex funnel equipped with a plastic shield, used to direct fumes up into the funnel, and capped at the tip with a layer of Parafilm^®^ M laboratory film (American National Can, Menasha, Wis). The funnel was manually held against the surrounding skin over and as close as possible to the excision site during the excision period. Immediately following this 2-minute collection period, a 10-mL-air sample was obtained from the apex of the funnel by puncturing the Parafilm using a 10-mL-glass air tight syringe, which was fitted with a 10.5-cm-long pipette tip, such that fumes from the upper portion of the funnel (beyond the stem) were sampled.

Analyses were conducted using a Minicams^™^ (OI Analytical, CMS Field Products Group, Birmingham, Ala; a combination preconcentrator tube and gas chromatograph with a flame photometric detector). The utilization of a Minicams^™^ to detect HD vapor over the skin of pigs has previously been described.^45^ Immediately before beginning these experiments, the Minicams^™^ was calibrated using standards of 1.9 ng/mL HD in hexane, which were prepared by dilution of stock agent that was provided at a concentration of 9.5 mg/mL HD in ethanol. The instrument was calibrated by injecting various volumes of the standard (1.5, 2.0, 3.0, and 4.0 µ *L*). The calibration was checked daily using injections of 3 µ *L* of the standard.

The Minicams^™^ is designed to collect HD vapor on a Tenax^™^ TA solid sorbent (Scientific Instrument Services, Ringoes, NJ), thermally desorb the bound agent, gas chromatographically separate mixtures, and detect HD using a flame photometric detector. The 3.67-minute (220-second) cycle of the Minicams^™^ consisted of a 1-minute-sampling period (0–60 s) during which a sample is collected on a preconcentrator tube containing Tenax^™^ followed by a 2.67-minute (60- to 220-second) purge when desorption and gas chromatographic analysis occurs. Each 10-mL-air sample that was collected from the Pyrex funnel was immediately injected over a 10-second interval during the 1-minute-sampling cycle into the Minicams^™^ through a heated vacuum line. The sample was drawn onto the preconcentrator tube within the Minicams^™^ at 0.7 L/min monitored by a mass flow meter (model FM-360, Tylan Corporation, Torrance, Calif). A 15-meter, 0.32-mm inner diameter, 5-µ m DB-1 column with a helium flow rate of 40 mL/min produced HD retention times of 138 seconds with a temperature program of initial temperature 50°C and a temperature ramp rate of 200°C/min to a final temperature of 200°C held for 115 seconds. The amount of agent detected in the sample was recorded both in nanograms and an 8-hour-time weighted average (TWA, where 3 ng/L HD = 1 TWA).

### Hexane extractions for HD

Unreacted (free) HD was extracted from the preexposure biopsies (series 1), presurgical absorbent swabs (series 2), excised wound tissue (series 3), and peripheral biopsies (adjacent and distant; series 4 and 5, respectively) by placing them in 50-mL aliquots of 99.9% pure Optima grade *n*-hexane (Fisher Scientific, Pittsburgh, Pa) for 24 hours at room temperature. After 24 hours, the extracted solutions were vortexed and samples transferred into autosampling vials in 1-mL aliquots in duplicate and stored at −80°C until further analysis.

Analytical separations were performed on an Agilent 6890 Plus GC-FID (Gas Chromatography—Flame Ionization Detector; Agilent Technologies, Wilmington, Del). An Agilent 7683 autoinjector was used for all injections. The entire GC system was controlled by Agilent Chemstation A.08.03 software (Agilent Technologies, Wilmington, Del) operating on a desktop computer. A 30-meter, 0.32-mm, 0.25-µ EC-1 capillary GC column (Alltech Associates, Deerfield, Ill) was used for all sample analyses. A split/splitless inlet was used in split mode at a 50:1 split ratio. The instrument was calibrated before measurements on each of the 5 series of samples. Calibration standards were prepared by 10:1, 5:1, and 2:1 volumetric dilutions of a primary 9.4 mg/mL HD in hexane gravimetric preparation. A fourth calibration standard was the primary 9.4 mg/mL preparation. Calibration curves were determined by the Agilent Chemstation A.08.03 software by averaging the peak areas for 3 injections of each of the 4 standards. Agilent Chemstation software was used to calculate concentrations of experimental samples on the basis of linear regression of the 4 calibration standards. Calibration curves, linear regression, and statistical calculations were made using GraphPad Prism plotting software, version 3.02 (GraphPad, San Diego, Calif).

### Statistical analyses

For the Minicams^™^ data, the amount of HD (ng) and TWA (8 h, stated as multiples of 3 ng/L) were analyzed by first testing for normality using a Shapiro-Wilk test. Having failed this test, the data were transformed using logarithms. The transformed data were found to be normally distributed. Comparisons of anatomical location (shoulder and thigh) and surgical tools (scalpel, Fugo Blade^®^, Bovie^®^, and Versajet^™^) were made using a mixed effects model analysis with tools as the fixed factor and locations as the random factor. Because there were some significant location differences, a 1-factor analysis of variance was performed for each location to compare tools, followed by a Tukey test to compare pairs of tools.

For the HD extraction data from the swabs and tissue samples, the amount of HD in the samples (as determined by the GC-FID) was analyzed. A comparison of tools and locations was made using a mixed effects model analysis with tools as the fixed factor and locations as the random factor. Accurate quantification below 0.1 mg/mL was not attempted, and therefore HD detected below this level was considered at one half that value (0.05 mg/mL) in the statistical analysis. Values were converted to milligrams for use in the analysis.

SPSS^®^ version 13.0 (SPSS Inc, Chicago, Ill) and StatXact (Cytel Inc, Cambridge, Massachusetts) were used to perform these analyses. Statistical significance was defined as *p* ≤ .05.

## RESULTS

### Minicams^™^ measurements

Plumes of smoke or vapor could be clearly seen emanating from the sites during excisions with both the Bovie^®^ electrosurgical knife and the Fugo Blade^®^. These fumes were successfully captured by the collection funnel. Although no visible plumes were noted with the scalpel blade or the Versajet^™^, the air above the operating field was sampled in the same way as it was during Bovie^®^ knife and Fugo Blade^®^ use.

**Figure 2:**
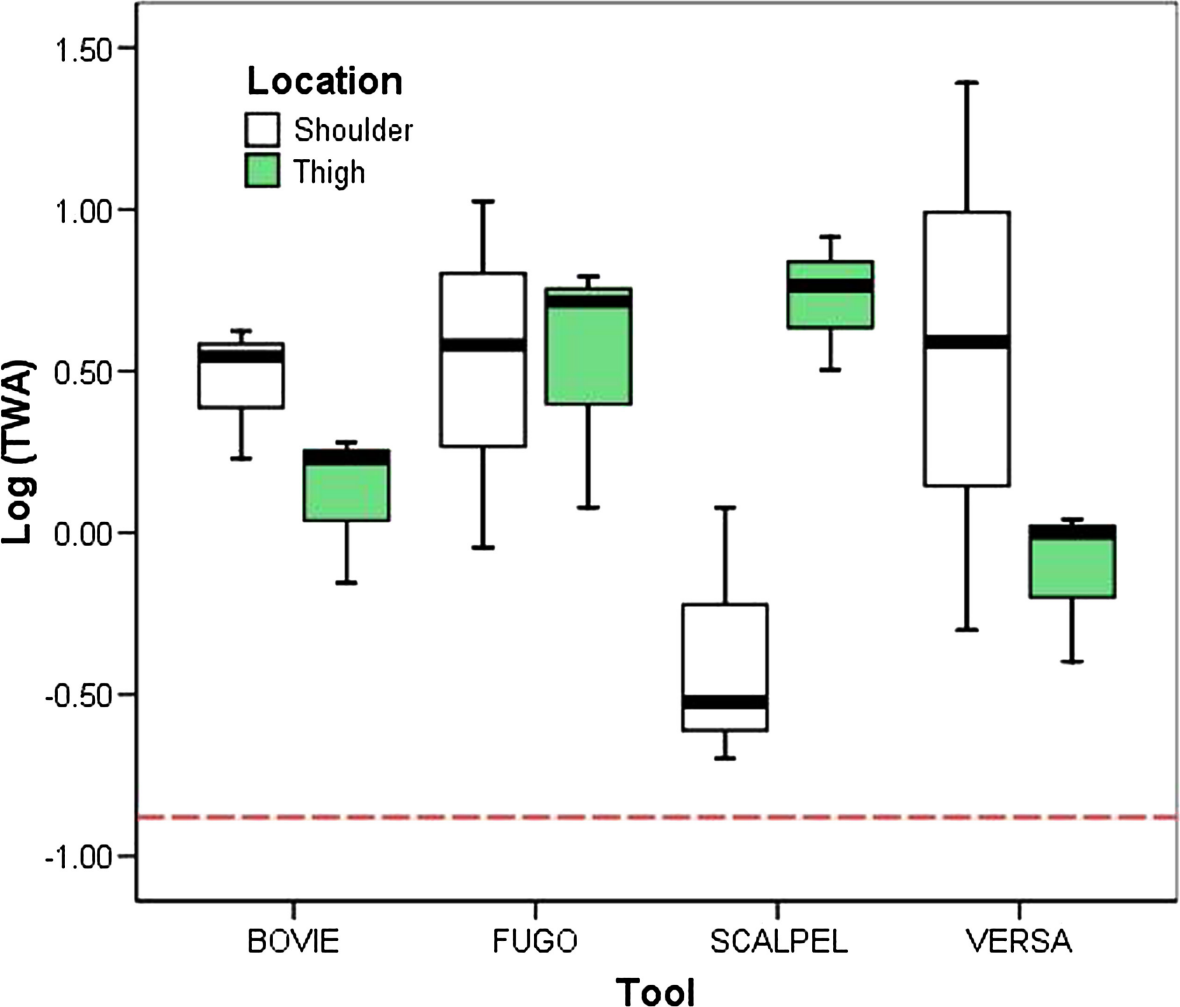
Log (TWA) by location and tool. A significant interaction of wound location (shoulder, thigh) and tool (no. 15 scalpel blade, Bovie^®^ knife, Fugo Blade^®^, and Versajet^™^) was observed. This interaction appeared to be due to the scalpel group. When the scalpel group was removed from the analysis, the interaction was not significant, and no other significant differences were observed between tools and locations. A separate analysis for each location was performed to compare tools. There were no significant differences observed among the tools for the shoulder wounds. A significant difference between the scalpel and Versajet^™^ was observed for the thigh, where the amount of HD detected in the collected fumes was significantly lower for the Versajet^™^ than for the scalpel blade. TWA = 8-hour-time-weighted average where 3 ng/L of liquid sulfur mustard = 1 TWA. The dashed red line represents the Department of Army's worker population limit 8-hour TWA of 0.0004 mg/m^3^. BOVIE = Bovie^®^ Electrosurgical Knife, FUGO = Fugo Blade^®^ M100 Anterior Capsulotomy Unit, SCALPEL = no. 15 scalpel blade, VERSA = Versajet^™^ Hydrosurgery System.

The data were log-normally distributed, and therefore, the analysis was conducted on the log transformed data. A significant interaction of wound location (shoulder, thigh) and tool (no. 15 scalpel blade, Bovie^®^ knife, Fugo Blade^®^, and Versajet^™^) was observed (Fig 2). This interaction appeared to be due to the scalpel group. When the scalpel group was removed from the analysis, the interaction was not significant and no other significant differences were observed between tools and locations.

A separate analysis for each location was performed to compare the surgical tools due to the interaction and some significant differences observed between locations. There were no significant differences observed among the tools for the shoulder wounds. A significant difference between the scalpel and Versajet^™^ was observed for the thigh, where the amount of HD detected in the collected fumes was significantly lower for the Versajet^™^ than for the scalpel blade. All measured levels significantly exceeded established safety limits.

### Hexane extractions for HD

Hexane extractions were performed on preexposure biopsies, presurgical absorbent swabs, excised wound tissue, and peripheral biopsies (adjacent and distant). The amount of unreacted (free) HD in each sample was determined by GC-FID (Table 1).

No HD was detected in the presurgical biopsies or peripheral biopsies collected distally to the surgical margins for any tools or at either location. Liquid sulfur mustard was detected in a single peripheral biopsy set taken from the thigh of 1 pig, adjacent to the surgical margin following wound excision with the Fugo Blade^®^.

Liquid sulfur mustard was consistently measured from the extraction solutions of the presurgical swabs; however, there were no significant differences between locations and tools. Of all samples evaluated, these swabs contained the highest amounts of HD (60% of the applied dose, on average).

Liquid sulfur mustard was extracted from the excised wounded tissue (1% of the applied dose, on average). There were no significant differences observed between locations or tools.

## DISCUSSION

This study has demonstrated that simple excisional debridement, regardless of the type of instrument used, can be very effective in HD wound decontamination. This study scope was limited to full-thickness skin wounds and constitutes a step forward in understanding how to manage agent-contaminated traumatic wounds. A logical follow-on would be systematic investigation of penetrating wounds in other locations involving relevant mechanisms. In the area of mitigating agent release from the wound during debridement, the Versajet^™^ was most promising, with significantly lower levels of HD detected over the thigh wounds compared with the scalpel-blade-treated wounds. It is unclear why there was a difference with this tool depending upon the location where it was used.

An important initial wound management issue raised in this study was the role of agent dilution within the wound versus block excision of the contaminated wound site. The 3 cutting tools, scalpel, Bovie^®^ knife, and Fugo Blade^®^, all demonstrated successful block excision technique. Liquid sulfur mustard was detected in a single peripheral biopsy set taken from the thigh adjacent to the surgical margin following wound excision with the Fugo Blade^®^, but this was likely due to surface contamination of agent rather than to lateral spread of HD below the skin surface. Block excision technique produces less hazardous waste and may be used to support histopathological, forensic, and attribution efforts. The Versajet^™^ performed well as a debridement tool and demonstrated that it could also irrigate and safely remove lavage fluid from the contaminated wound site. It also generated the greatest amount of hazardous waste, the majority being lavage fluid mixed with contaminated tissue homogenate. Because this tool can confine its lavage action over a small area, it may actually promote more efficient lavage, thereby conserving limited supplies. Depending on the pressure setting used and how the hand piece is moved, the instrument can be used either as a cutting tool for making incisions and cutting away tissue or to remove tissue and debris in a tangential plane. Future study needs to investigate these capabilities. Its utility may be limited to mature medical facilities with robust infrastructure due to its logistical footprint, which includes wall power, large fluid stockpile, and hazardous waste recovery and disposal.

This study also added some insight into agent activity around the wound site. There was no measurable lateral spreading of the agent beyond the surgical margins. According to the conventional wisdom, HD should have been rapidly fixed to tissue components,^46^ quickly absorbed by the lipophilic subcutaneous fat, absorbed systemically, and/or spread along fascial planes. This did not completely occur in this animal model as witnessed by the significant amount of agent that could be swabbed out of these wounds 60 minutes after exposure, immediately before excision. The existence of a dermal reservoir of HD in humans was first suggested in World War I by Smith et al,^47^ who demonstrated that HD injuries could be prevented by washing contaminated skin with an appropriate solvent up to 45 minutes postexposure. Furthermore, Smith et al demonstrated that the skin reservoir of HD could be transferred to a second individual, even after the exposed surface had been decontaminated. However, studies conducted during World War II reported the opposite effect in that HD was rapidly “fixed” by skin constituents such as proteins (Renshaw^48^). Contemporary in vitro studies have confirmed the original work of Smith et al that a substantial reservoir of HD is formed in human skin that can account for up to 35% of the applied dose after 24 hours.^49^ Work is under way to further examine the toxicology of this reservoir in both pig and human skin exposed to radiolabeled HD ex vivo. Studies are being conducted to determine the exact location of this reservoir of unreacted agent, its persistence and kinetics, the diffusional resistance of the main skin layers (stratum corneum, epidermis, dermis, hypodermis) to this agent, the spreading characteristics of HD on the skin surface, and physical and chemical techniques in removing the depot. The existence of the agent reservoir has implications for the safety of medical emergency personnel treating HD casualties with or without concurrent trauma. In addition to safety-related implications, the existence of such a depot may exacerbate cutaneous and possibly systemic injury if the exposed site is occluded by clothing or semiocclusive/occlusive dressings, thereby allowing the agent to further penetrate into the skin rather than off-gas into the atmosphere. Verification of a depot phenomenon would lend further justification to far-forward topical or surgical decontamination as a legitimate means to contain this hazard.

As stated earlier, both the Bovie^®^ knife and the Fugo Blade^®^ produced observable plumes during use. The use of electrocautery, common in surgical practice, has the potential to increase the risk of exposure to HD vapors by heating up the tissue and volatizing any unreacted (unbound) agent. This study is the first to observe the use of low-power plasma to contain and manage agent wound contamination with Fugo Blade^®^ use. This tool produced a visible plume of vapor during the excision procedures. Unlike diathermy, which produces pungent thermal oxidative fumes or residue (“smoke”), the Fugo Blade^®^ produces a plume composed of vaporized water laced with aromatic molecular fragments and is similar to the laser plume generated by excimer laser ablation of the corneal surface.^32^,^50^ As the physical basis of plasma sterilization techniques, microbes cannot exist in the plasma cloud generated by the Fugo Blade^®^ (Swarthmore College Plasma Lab, Swarthmore, Pa, unpublished data, August 2000). This observed microbe decontamination effect was the basis for preliminary investigation of the plasma cloud's effect on smaller chemical agent molecules such as HD. Earlier work by Herrmann et al of Los Alamos National lab showed that small area chemical agent decontamination was achievable by using nonthermal, atmospheric pressure, low-temperature plasma.^51^,^52^ Although initially developed for nonmedical applications, recent advancements have produced decontaminating plasma streams at operating temperatures consistent with possible intraoperative use (Dr Gary S. Selwyn, personal communication, Los Alamos National Laboratory, Los Alamos, NM). This basic concept of a decontaminating plasma field was applied to the problem of containing or neutralizing agent liberated into the air over the contaminated wound during operation, using the excising plasma field as a mitigation tool for volatized HD agent. In this study, no significant reduction of HD was detected over the operating field. Although explanation of this observation is conjectural, one possible explanation is that the M100 plasma cloud was too small for both efficient cutting and localized decontamination. The device used in this study was a microsurgical device used beyond its design parameters; the study operating team noted that the M100 microsurgical device was less efficient in cutting in this application than the other modalities used in this study. A new Fugo Blade^®^ designed for use in general surgical applications, such as excisional debridement, was not available at the time of this study and warrants additional research. This new system retains the desired attributes of the original Fugo Blade^®^ M100 system. As both the Bovie^®^ knife and the Fugo Blade^®^ were also used to control bleeding during surgery, the use of hemostats, pressure, epinephrine-soaked pads, and other adjuncts may be better alternatives to attain hemostasis in this setting.

There is an obvious need for an effective nontoxic decontaminant that is safe to use in the eyes, open wounds, and body cavities. Although aggressive flushing may prove beneficial, a decontaminant that both extracts and neutralizes a wide variety of CWAs (both nerve and vesicating agents) would be ideal. Currently available topical skin decontamination systems such as Reactive Skin Decontamination Lotion (RSDL,^53^ E-Z-EM Inc, Lake Success, NY), the US military's M291 Skin Decontamination Kit (U.S. Army Tank—Automotive and Armaments Command, Rock Island, Ill) with Ambergard XE-555 resin^46^,^54^ and fuller's earth are all contraindicated for use as wound decontaminants. In the case of RSDL, this is due in part to the potential systemic toxicity of an active ingredient (2,3-butanedione monoxime).^55^ An additional drawback to RSDL use within wounds is the evidence that it impairs wound strength and decreases collagen content in the early phases of wound healing.^56^ Topical decontaminants based on fine powders such as fuller's earth and M291 may cause localized toxicity. They are difficult to remove from wounds, and residual particles may cause chronic irritation, resulting in fibrosis and a granulomatous reaction in surrounding tissue.^57^,^58^ In addition, some topical decontaminants employ indicator systems to show where decontaminant has been applied, which in turn may obscure the surgeon's visibility if used inside a wound. The ideal wound decontaminant would also be hemostatic; no product thus far has shown this property. Plans for development of a hemostatic agent that can concurrently extract and decontaminate CWAs (vesicants or nerve agents) are under way.

Battlefield wounds are commonly contaminated with dirt, shrapnel, clothing, blast debris, bone dust, wood, or concrete fragments that are driven into the wound and require judicious lavage and timely debridement of devitalized tissue.^5^,^13^ Wounds contaminated with CWA should also be well irrigated before excisions or debridement procedures are conducted. The use of freshly prepared dilute hypochlorite (0.5%) as a skin decontaminant for use in deep, noncavity wounds has been suggested but is contraindicated for corneal, brain, and spinal cord injuries.^46^ Irrigation of the abdominal cavity with hypochlorite solution may induce adhesions, and the danger of its use in the thoracic cavity is unknown.^46^ Saline or other surgical solutions may be used for irrigation; however, the run-off effluent should be considered potentially contaminated^46^ and could spread the CWA throughout open cavities. Such fluids should not be removed with surgical sponges, as contaminated sponges will not decontaminate the agent and may spread any contamination present. Instead, these fluids should be removed by suction. Flushing noncavity wounds with copious quantities of saline before surgical intervention may prove beneficial if unintended spread of the CWA can be controlled. The use of a pressure wound irrigation device with splatter guard is recommended to provide optimal pressure of saline and prevent backsplash. The Versajet^™^ may be the ideal tool to use because it can be used to irrigate and safely remove lavage fluid from the wound site even before surgical excision.

Finally, the hazard posed to personnel during exposure to an HD-contaminated wound may be understated and widely misunderstood. A 10-µ g droplet of HD is enough to cause vesication. The threshold for vapor/aerosol to induce cutaneous damage is 200 to 2000 mg-min/m^3^ (*Ct*), dependent on the anatomical location, environmental conditions (temperature and humidity), sweating, and other factors.^59^ For ocular damage, the *Ct* is 12 to 70 mg-min/m^3^ under field conditions. The estimated *Ct* for airway injury is 100 to 500 mg-min/m^3^. The toxicology, clinical manifestations, pathogenesis of injury, and long-term effects of HD have been previously summarized.^59^,^61^ In this experiment, vesicating levels of HD could be extracted from the excised tissue. The average amount of HD (95% confidence interval) extracted from all excised tissues was 9.9 mg (6.2 mg, 13.7 mg). This is 1000 times the amount necessary to induce vesication (with a 10-µg topically applied droplet, about 80% of this evaporates from the skin surface, 10% enters the circulation, leaving about 1 µg to produce vesication^59^. Any tissue handled or removed during damage control surgery thus poses a hazard to medical personnel. The duration of this hazard is unclear. For HD-contaminated tissue, decontamination could be accomplished by first cutting the excised tissue into small pieces to increase contact surface area and then submerging the pieces for extended soaking in household bleach (5% hypochlorite) that is periodically refreshed and agitated. If nondestructive tissue handling is required, as in follow-on histopathological examination for forensics or attribution, no proven alternatives to bleach have been demonstrated and further study in this area is needed. All tissue samples harvested from presumptively contaminated wounds should be carefully handled and considered potentially hazardous. Although it is clear that HD was absorbed in the wound cavities, some of which was extractable with an organic solvent, it is unknown where this agent was residing within the tissue. The use of radiolabeled agent and microautoradiography or confocal Raman spectroscopy may be useful in pinpointing the exact location of agent contamination in open wounds. It is unclear how long unreacted agent will reside in open wounds. Ex vivo studies to examine the existence, kinetics, and time course of a reservoir of unreacted HD in pig and human skin are nearing completion and results will be reported soon.

The *Textbook of Military Medicine*^46^ and the 2004 edition of *Emergency War Surgery* state that there is no vapor release from contaminated wounds without foreign bodies and off-gassing from a wound during surgical exploration will be negligible or zero. This was not found to be the case in this study. Before this study, wound healing studies using weanling swine have demonstrated that following topical exposure to undiluted liquid HD for 120 minutes, there was a significant period of off-gassing of unbound agent, as measured by a Minicams^™^ (Graham et al, unpublished results). Although the textbook states that a chemical protective mask is not required for surgical personnel, the data from this and preceding studies indicate a present risk, and appropriate personal protection should be utilized, especially in a battlefield arena where CWA use has been detected or suspected. In this study, a significant amount of HD was extracted from the wound swabs taken immediately before surgery (60% of the applied dose, on average). The majority of the applied agent remained in the biopsy cavities and was not immediately absorbed by the subcutaneous fat or walls of the open wound as anticipated. Although approximately 1% of the applied dose was extractable from the excised tissue, it is unclear how much of the unaccounted for remaining agent had evaporated before swabbing, became fixed to tissue components, or was absorbed systemically. Because of the low volatility of HD and the limited surface area on which the applied HD was exposed to air, the amount of applied HD lost to evaporation was likely very small. This small amount of HD vapor was detected above the surgical field for each of the 4 tools. In general, there was no difference in the amount of HD detected among the tools used. The amounts of HD vapor were, on average, 29 times higher than the Department of Army's worker population limit 8-hour TWA of 0.0004 mg/m^3^ and 4 times higher than the 15-minute short-term exposure limit of 0.003 mg/m^3^. Taking into account the threshold limits for exposure noted above if a surgical staff works unprotected for many hours to treat multiple patients with contaminated wounds in a relatively small enclosed area such as Chemically and Biologically Protected Shelter Systems,^7^,^12^ their risk of ocular or airway damage from the vapors may be significant. Absent verifiable decontamination, appropriate protective measures should be taken, which should include use of a chemical-biological mask and smoke evacuator such as those used during laser debridement or resurfacing procedures. Guidance on the use of personal protective gear including masks and chemical protective gloves can be found in the *Textbook of Military Medicine*.^46^,^54^ Dexterity while wearing loose-fitting chemical protective gloves can be greatly enhanced by wearing an overlying pair of appropriately sized surgical gloves.

This risk has particular relevance in today's casualty management system: injured warfighters currently stationed in Iraq are evacuated to medical care at a CSH on average 30 to 60 minutes from the time of injury.^9^,^11^ Surgical critical care by the forward surgery teams or at a CSH begins within 1 to 4 hours after injury following transport by ground or air evacuation.^12^ During this time frame, any CWAs contaminating the wound, especially HD, may not have been completely absorbed into the wound or completed their off-gassing phase, thus posing a threat to attending caregivers. In addition, agent that has been absorbed may not have completely reacted with tissue components or been degraded and may pose an additional threat to both the patient and those handling the tissue.

An attempt was made with this study to point a way forward for surgical decontamination by agents with latent or delayed effects, but why is this needed, from a military perspective? Sustainment of warfighting capability in the face of chemical, biological, radiological, and nuclear weapon employment is a vital command concern. Employing specialized expertise to fight within this environment is a ready solution but has profound limitations. Containment of chemical, biological, radiological, and nuclear agent effects is another readily available remedy. Early elimination of contaminated tissue “contains” the hazard, thus possibly expediting movement and improving casualty survival or loss of function; these casualties can then flow into the matured care and movement system that serves casualties suffering conventional combat injuries. Advances in regenerative medicine and prosthetics may mitigate effects of early surgical measures to resuscitate, control damage, and decontaminate. Further research into the use of an effective decontaminant that is nontoxic to tissue and can be used in open wounds and further experimentation with tools such as the Versajet^™^ and Fugo Blade^®^ for effective removal of CWA contaminants from wounds as well as debridement are warranted. These data provide insight for future studies to determine the best treatment of combined traumatic and CWA injuries for the warfighter and civilian injured during acts of war or terrorism.

## Figures and Tables

**Figure 1a:**
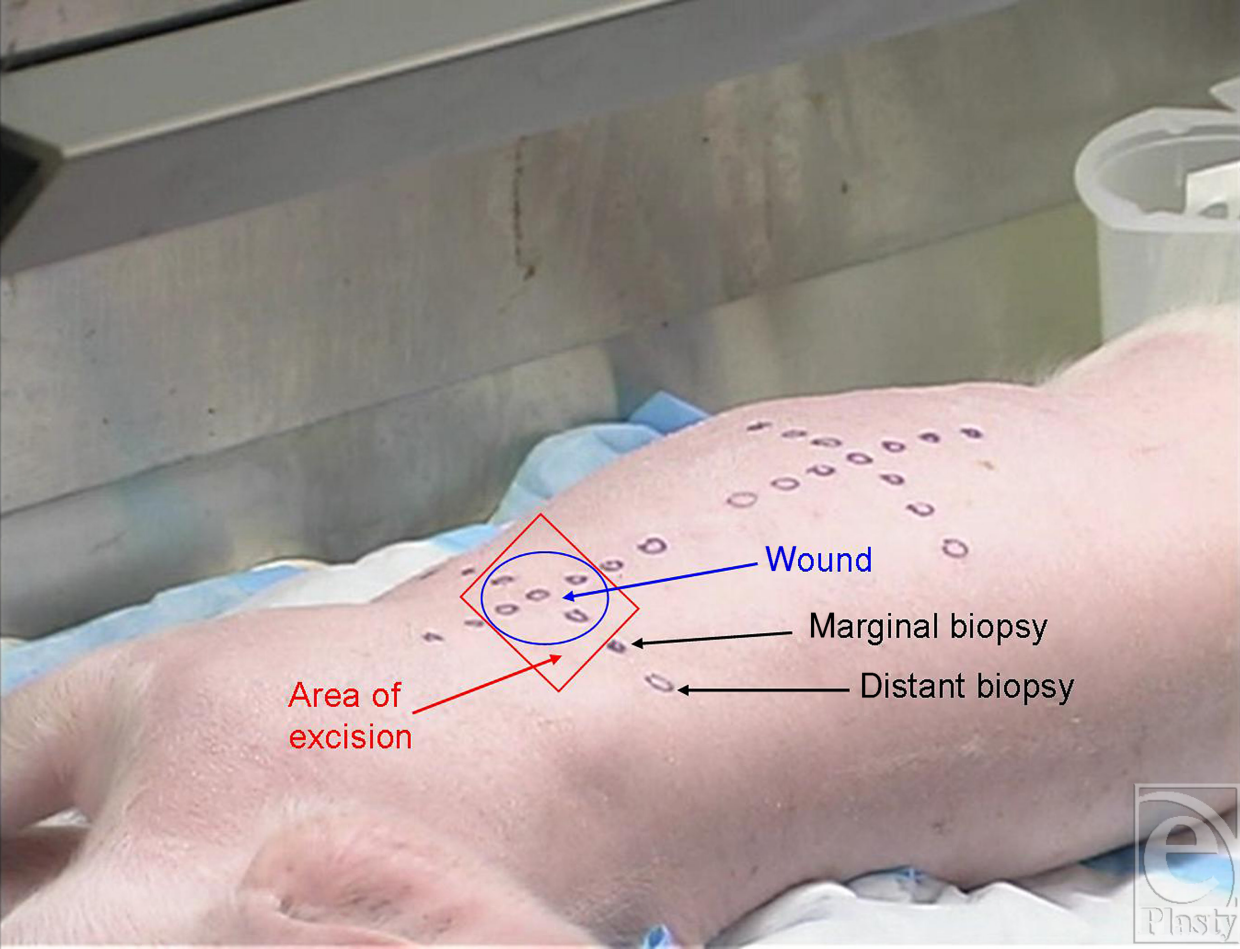
Preparation (preexposure template marking).

**Figure 1b:**
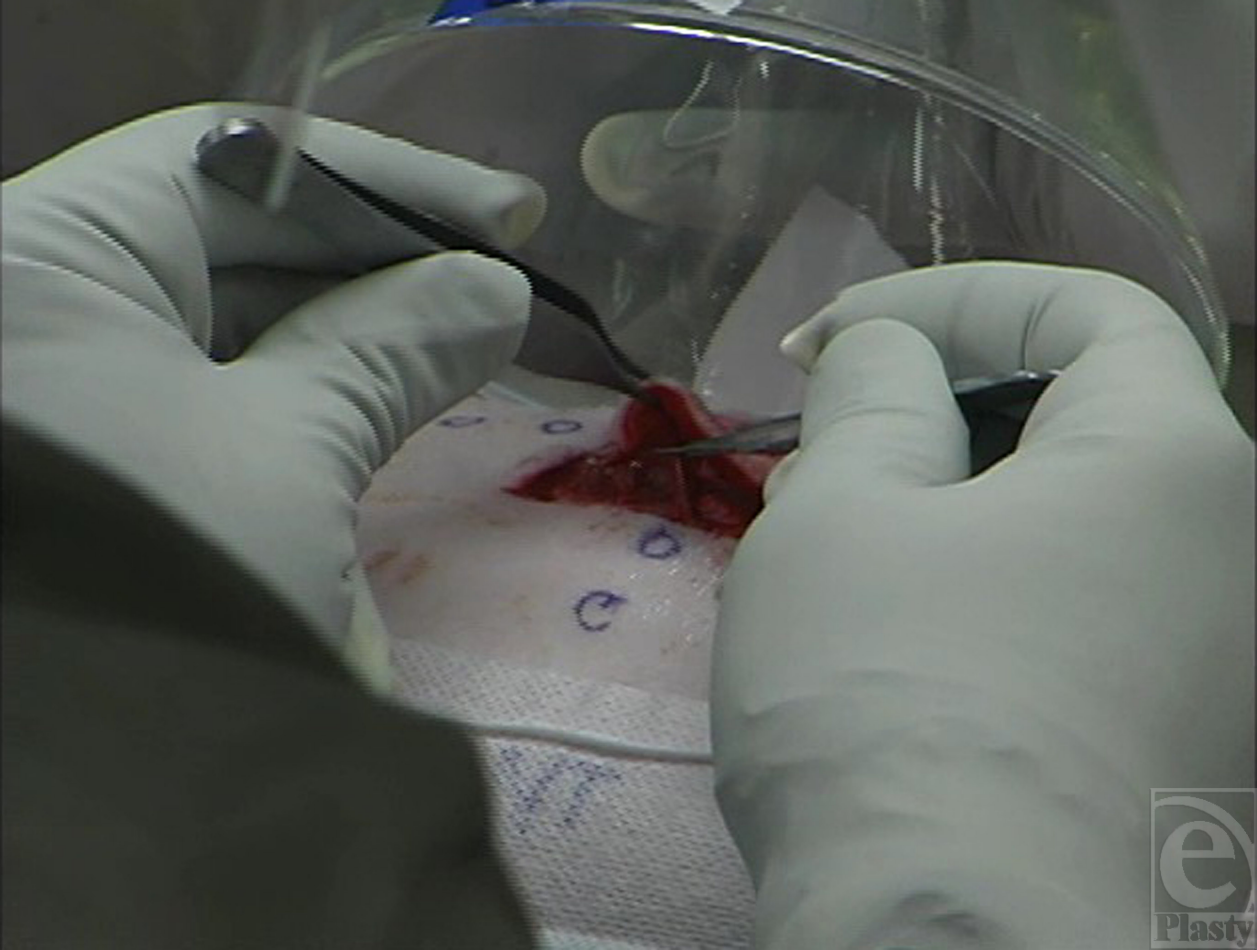
Scalpel excision with gas capture funnel over field.

**Figure 1c:**
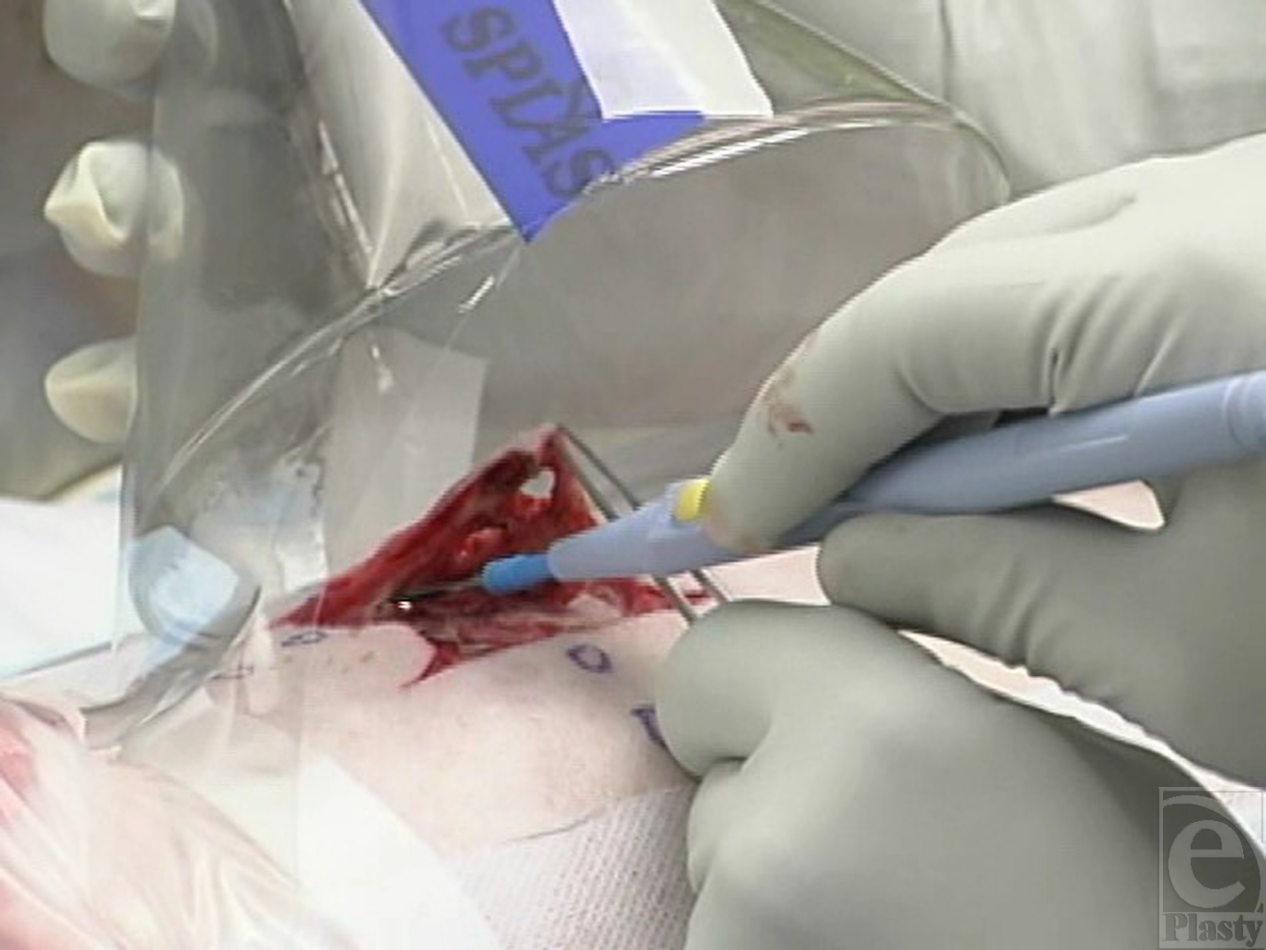
Bovie^®^ excision with gas capture funnel over field.

**Figure 1d:**
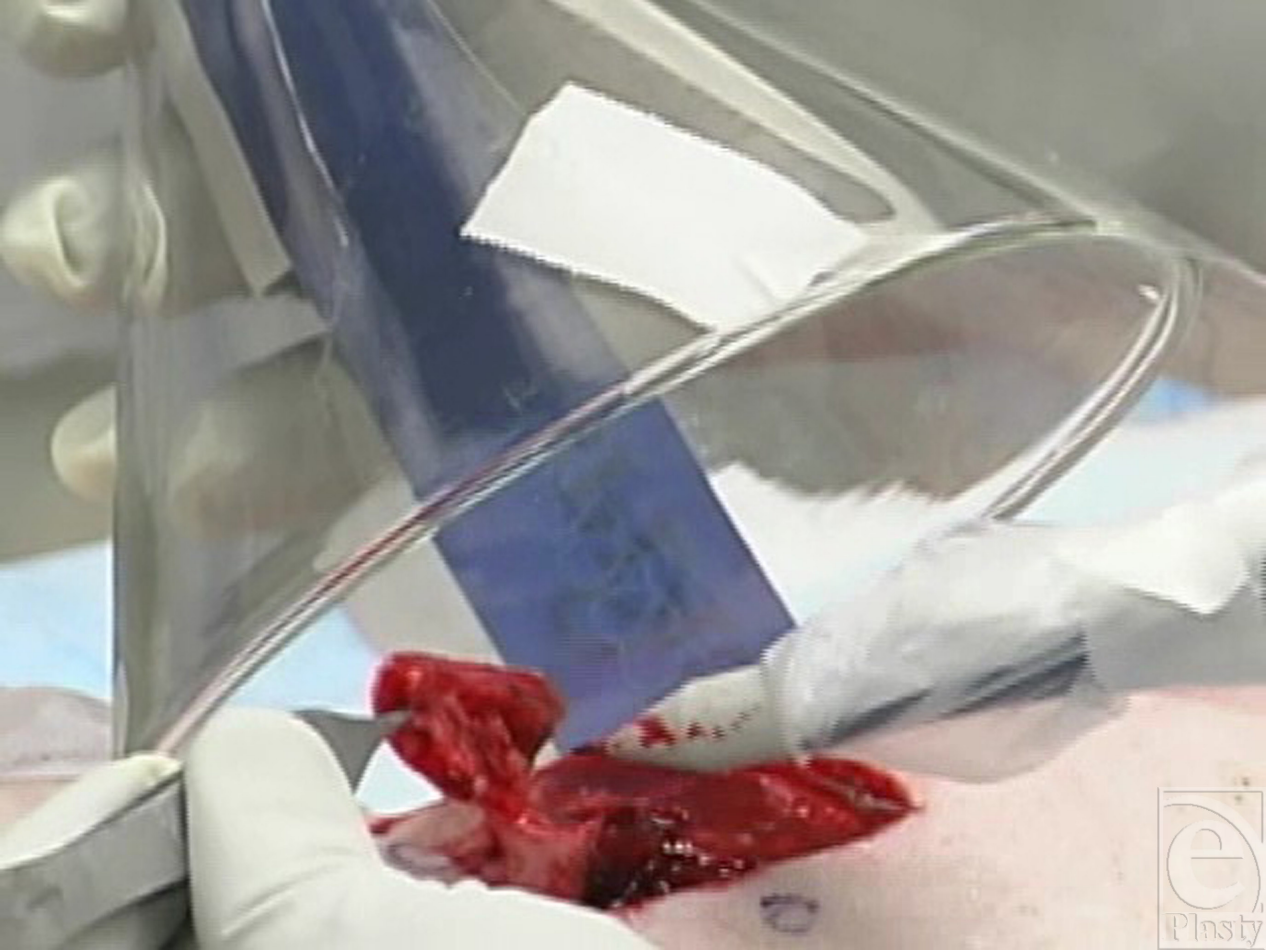
Fugo Blade^®^ excision with gas capture funnel over field.

**Figure 1e:**
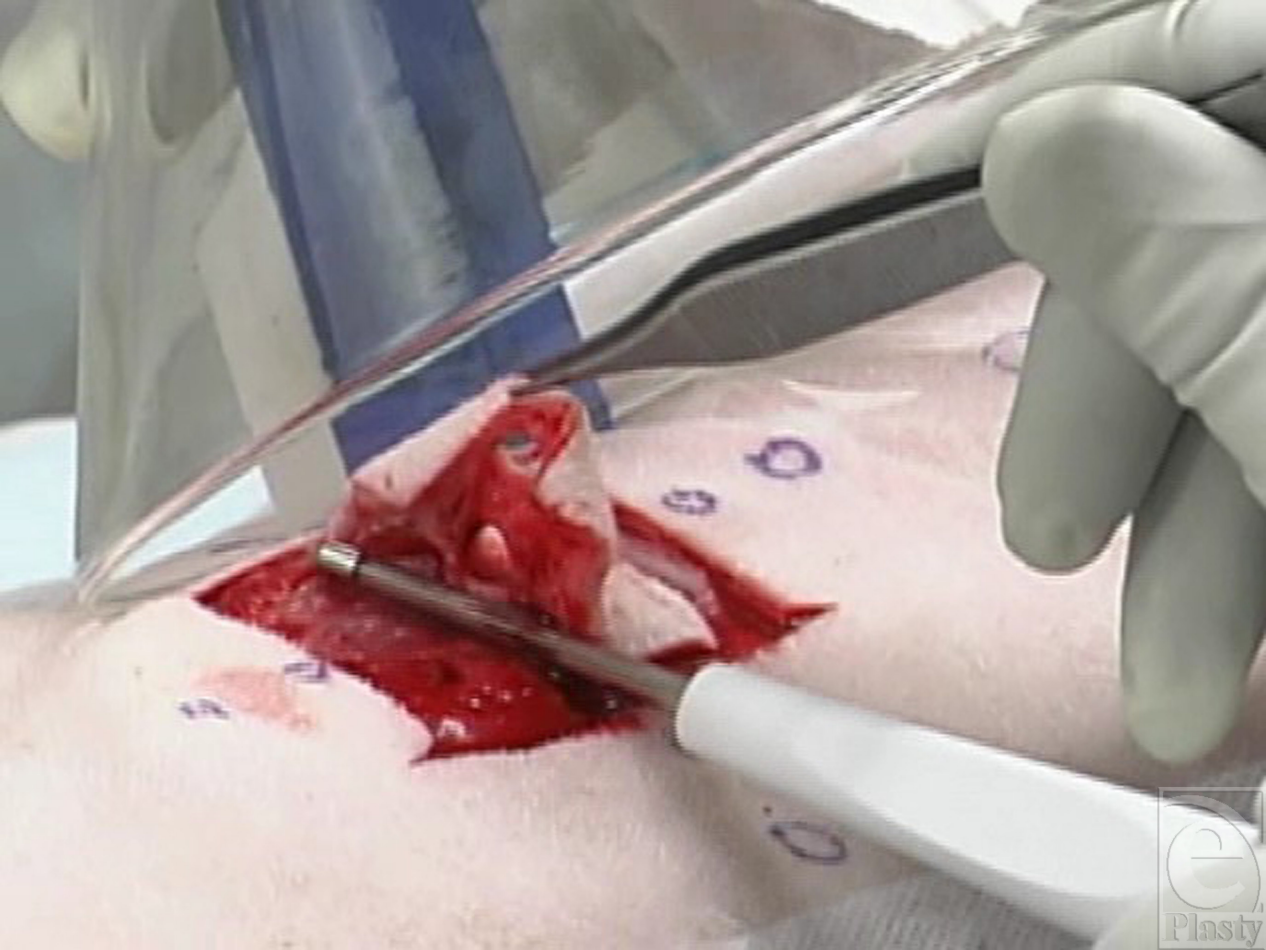
Versajet^™^excision with gas capture funnel over field.

**Table 1:** Unreacted (free) liquid sulfur mustard (mg) by location and tool

Location	Tool		Presurgical biopsies	Presurgical wound swabs	Excised tissue	Biopsies proximal to surgical margin	Biopsies distal to surgical margin
Shoulder	BOVIE	Mean	0.0	737.5	5.3	0.0	0.0
		SD	0.0	173.9	4.9	0.0	0.0
		Median	0.0	712.5	2.5	0.0	0.0
		*N*	3	3	3	3	3
	FUGO	Mean	0.0	492.5	20.2	0.0	0.0
		SD	0.0	121.5	30.6	0.0	0.0
		Median	0.0	430.0	5.2	0.0	0.0
		*N*	3	3	3	3	3
	SCALPEL	Mean	0.0	1027.5	13.3	0.0	0.0
		SD	0.0	385.4	12.2	0.0	0.0
		Median	0.0	1207.5	11.0	0.0	0.0
		*N*	3	3	3	3	3
	VERSA	Mean	0.0	924.2	7.5	0.0	0.0
		SD	0.0	133.3	1.5	0.0	0.0
		Median	0.0	907.5	7.5	0.0	0.0
		*N*	3	3	3	3	3
Thigh	BOVIE	Mean	0.0	573.3	1.7	0.0	0.0
		SD	0.0	239.5	1.4	0.0	0.0
		Median	0.0	520.0	2.5	0.0	0.0
		*N*	3	3	3	3	3
	FUGO	Mean	0.0	752.5	4.0	3.2	0.0
		SD	0.0	173.3	2.6	5.5	0.0
		Median	0.0	657.5	2.5	0.0	0.0
		*N*	3	3	3	3	3
	SCALPEL	Mean	0.0	885.0	17.3	0.0	0.0
		SD	0.0	152.8	18.8	0.0	0.0
		Median	0.0	875.0	8.0	0.0	0.0
		*N*	3	3	3	3	3
	VERSA	Mean	0.0	605.0	9.8	0.0	0.0
		SD	0.0	187.6	12.0	0.0	0.0
		Median	0.0	690.0	6.2	0.0	0.0
		*N*	3	3	3	3	3

*Note*. BOVIE, Bovie^®^ electrosurgical knife; FUGO, Fugo Blade^®^ M100 Anterior Capsulotomy Unit; SCALPEL, no. 15 scalpel blade; VERSA, Versajet^™^Hydrosurgery System.
